# Standardized Mean Differences: Not So Standard After All

**DOI:** 10.1002/cl2.70056

**Published:** 2025-08-17

**Authors:** Juyoung Jung, Ariel M. Aloe

**Affiliations:** ^1^ Educational Measurement and Statistics University of Iowa Iowa City Iowa USA

**Keywords:** coefficient of variation, data harmonization, effect sizes, meta‐analysis, standardized mean differences

## Abstract

Meta‐analyses often use standardized mean differences (SMDs), such as Cohen's *d* and Hedges' *g*, to compare treatment effects. However, these SMDs are highly sensitive to the within‐study sample variability used for their standardization, potentially distorting individual effect size estimates and compromising overall meta‐analytic conclusions. This study introduces harmonized standardized mean differences (HSMDs), a novel sensitivity analysis framework designed to systematically evaluate and address such distortions. The HSMD harmonizes relative within‐study variability across studies by employing the coefficient of variation (CV) to establish empirical benchmarks (e.g., CV quartiles). SMDs are then recalculated under these consistent variability assumptions. Applying this framework to Meta‐analytic data reveals the extent to which (original) effect sizes and pooled results are influenced by initial, study‐specific standard deviations to standardize mean differences. Furthermore, the method facilitates the inclusion of studies lacking reported variability metrics into the sensitivity analysis, enhancing the comprehensiveness of the meta‐analytic synthesis.

Synthesizing the effects of interventions, treatments, curricula, or programs (henceforth, treatments) has become common practice to inform policies and best practice guidelines. When the outcome under study is continuous (e.g., a student's exam score), the treatment effect can, in its simplest form, be estimated as the mean difference between treatment conditions. If all studies use the same instrument to measure the outcome, the treatment effect across studies can and should be compared using the original metric (Bond et al. 2003). However, study results are often standardized because outcomes are frequently reported using similar but distinct measurement scales, necessitating standardization to remove scale differences and enable cross‐study comparisons.

To facilitate comparison, the standardized mean difference (SMD) is used. The SMD, most commonly estimated using Cohen's *d* (Cohen 1969) and Hedges' *g* (Hedges 1981), is estimated by dividing the observed mean difference by the pooled sample standard deviation. However, the use of these measures has faced scrutiny in fields such as statistics, epidemiology, and public health. Among other limitations, a key concern is that standardized treatment effects are influenced by within‐study standard deviation variability, beyond representing mean differences. Consequently, SMDs may yield effect sizes that appear smaller or larger than those derived from comparisons using an original, common metric, primarily due to variations in the standard deviation (Ades et al. 2015; Bond et al. 2003; Greenland et al. 1991; Greenland et al. 1986; Rothman et al. 2008).

The concern over standardizer (i.e., study‐specific standard deviations used for standardization) variability deepens when considering that reported sample standard deviations are influenced by factors beyond true population heterogeneity. Idiosyncratic sampling, notably convenience samples, may yield standard deviations unrepresentative of broader population dispersion (Mercer et al. 2017). Study design elements (e.g., restrictive eligibility criteria, instrument‐specific floor/ceiling effects) or varied experimental control can also artificially alter observed variance (Greenland et al. 1991). Furthermore, variance estimates from small samples are inherently unstable and less precise reflections of population variability (Kelley and Rausch 2006). These factors render sample standard deviations “noisy” or biased standardizers. This denominator variability directly translates into inconsistent SMD calculations, complicating comparability and potentially undermining the robustness of meta‐analytic conclusions if not critically evaluated.

To illustrate the profound impact of standardizer variability, consider a hypothetical example where two studies, using identical instruments and methodologies, both report a mean treatment difference of 10 points. If Study 1 utilizes a pooled standard deviation of 2, its SMD is 5 $\left(i.e.,\frac{10}{2}\right)$. Conversely, if Study 2, despite the same mean difference, employs a pooled standard deviation of 1, its SMD becomes 10 $\left(i.e.,\frac{10}{1}\right)$. This doubling of the standardized effect, stemming solely from differing sample standard deviations, exemplifies how conventional SMDs (e.g., Cohen's d and Hedges' g) are highly sensitive to within‐study variability. While true heterogeneity in treatment effects across studies is a distinct and critical consideration, the present focus is on how the standardization process itself, intended merely to remove the original outcome scale, can inadvertently alter perceived effect magnitudes due to the instability of the sample standard deviation used as its basis.

This study addresses the need to critically evaluate the impact of within‐study standard deviation variability on SMDs by proposing the harmonized standardized mean difference (HSMD) framework. Designed as a sensitivity analysis for meta‐analyses using SMDs, HSMD examines the influence of varying study‐specific standard deviations by employing the coefficient of variation (CV) to quantify relative, scale‐independent variability and establish empirical benchmarks (e.g., data set‐derived quartiles) for recalculating SMDs with harmonized denominators. Comparing meta‐analytic results from HSMD sets to those from conventional SMDs enables assessment of the robustness of conclusions to standardizer variability. To demonstrate its utility, this study reviews SMDs, discusses relevant sampling variability, outlines the HSMD methodology, presents illustrative applications, and explores alternative harmonization approaches, concluding with practical implications.

## 1 SMDs

Consider a population regression model with one dichotomous variable (X) representing treatment conditions and a continuous outcome. This model can be expressed as $Y_{j}=\beta_{0}+\beta_{1}X_{j}+e_{j}$, where $Y_{j}$ is the continuous outcome for the jth individual, $\beta_{0}$ is the intercept, $\beta_{1}$ is the regression coefficient (slope) associated with X, and $e_{j}$ is the error term, assumed to be normally distributed, $e_{j}∼N(0,\sigma_{\text{pooled}}^{2})$. In this model, assuming homogeneity of variance across groups, the mean squared error serves as an estimator for the population pooled variance, $\sigma_{\text{pooled}}^{2}$.

The corresponding sample pooled standard deviation, $\sigma_{\text{pooled}}$, is calculated as ${\hat{\sigma }}_{\text{pooled}}=\sqrt{\frac{(n_{T}-1)s_{T}^{2}+(n_{C}-1)s_{C}^{2}}{n_{T}+n_{C}-2}}$, where $n_{T}$ and $n_{C}$ are the sample sizes, and $s_{T}^{2}$ and $s_{C}^{2}$ are the sample variances of the treatment and control groups, respectively. This ${\hat{\sigma }}_{\text{pooled}}$ is a sample‐based estimate of the underlying population pooled standard deviation, $\sigma_{\text{pooled}}$. Cohen's d, an estimate of the population SMD, is then defined as

(1) $$d=\frac{{\hat{\beta }}_{1}}{{\hat{\sigma }}_{\text{pooled}}},$$

where ${\hat{\beta }}_{1}$ is the estimated mean difference and ${\hat{\sigma }}_{\text{pooled}}$ is the specific pooled standard deviation from the sample used for standardization.

The approximate variance of d is given by (Egger et al. 2008; Hartung et al. 2011; Hedges 1981)

(2) $$\text{var}(d)=\frac{n_{T}+n_{C}}{n_{T}n_{C}}+\frac{d^{2}}{2(n_{T}+n_{C}-2)},$$

where the denominator of the second term in Equation (2) is occasionally written as $2(n_{T}+n_{C})$. As noted by Lin and Aloe (2021), among others, the difference between $2(n_{T}+n_{C}-2)$ and $2(n_{T}+n_{C})$ arises from differing assumptions in large sample approximations. Furthermore, Borenstein et al. (2009) observed that these two forms yield very similar results unless the sample sizes are particularly small.

The exact variance of d can be expressed as

(3) $$\text{Var}(d)=\left(\frac{n_{T}+n_{C}}{n_{T}n_{C}}\right)\left(\frac{m}{m-2}\right)\left(1+\frac{n_{T}n_{C}}{n_{T}+n_{C}}d^{2}\right)-\frac{d^{2}}{c{\left(m\right)}^{2}},$$

where c(m) represents the small sample bias correction factor. An approximation for c(m) is

(4) $$c(m)\approx 1-\frac{3}{4m-1},$$

where m=N−C−1 is the degrees of freedom (typically $n_{T}+n_{C}-2$ for a two‐group comparison), N is the total sample size, and C is the number of independent variables in the model. Hedges (1981) introduced this small sample bias correction for d. While the approximate formula for c(m) is commonly used in practice, the exact formula is

(5) $$c(m)=\frac{\Gamma \left(\frac{m}{2}\right)}{\sqrt{\frac{m}{2}}\Gamma \left(\frac{m-1}{2}\right)},$$

where Γ(⋅) is the gamma function and m represents the degrees of freedom, as previously defined.

Applying this small sample bias correction yields Hedges' g as

(6) g=d×c(m).

The rationale for estimators such as d and g is to standardize the observed mean difference (${\hat{\beta }}_{1}$) using a pooled standard deviation. Ideally, this standardizer should reflect the inherent variability of the outcome in the population of interest, without being artificially diminished by covariates included in the primary study's model. However, the extent to which the sample‐based ${\hat{\sigma }}_{\text{pooled}}$ accurately estimates the true population variability, $\sigma_{\text{pooled}}$, critically depends on the characteristics of the sample variances ($s_{T}^{2}$ and $s_{C}^{2}$) from which it is derived.

The variance of g is then

(7) $$\text{var}(g)=\text{var}(d)\times {\left[c(m)\right]}^{2}.$$

When computing SMDs, the pooled standard deviation serves as an estimate of the population's standard deviation, reflecting variability without adjusting for covariate effects. However, in practice, non‐probabilistic sampling (e.g., convenience samples) often means that differences in sample standard deviations may not solely reflect sampling error, potentially leading to inconsistent SMD estimates across studies (Mercer et al. 2017). Implementing the above formulas is straightforward, but researchers must carefully select the numerator and denominator in Equation (1). The numerator requires choosing the most appropriate representation of the treatment effect, which depends on the primary study's design and statistical model, often involving adjusted mean differences. The denominator, an estimate of the population's standard deviation, directly influences the SMD metric.

### 1.1 Variability and Sampling

When a target population is well‐defined, ideal random samples drawn from it are representative subsets of this larger population. In such cases, differences observed in sample standard deviations across studies can primarily be attributed to inherent sampling variability. If samples are probabilistically drawn (i.e., random samples), they are considered unbiased representations of the larger population from which they originate. Variations among these random subset samples are thus expected due to chance. The magnitude of such expected variation among different random samples is influenced by several factors, including the overall population size, the individual sample sizes, the natural variability within the population itself, and the specific sampling method employed (Lohr 2021).

However, when samples are not probabilistically drawn from a population, there is no inherent guarantee that the observed sample variance will accurately reflect the true variability of the outcome in the target population. The variance of a convenience sample can deviate from the true population variance in unpredictable ways. By its nature, convenience sampling introduces non‐random selection processes and, consequently, potential biases (e.g., selection bias). For instance, if a convenience sample disproportionately includes specific subgroups with greater or lesser heterogeneity, this can inflate or deflate the sample's observed variability compared to the population. Similarly, if the sample inadvertently excludes individuals with extreme scores that are present in the broader population, the resulting sample variance might be artificially diminished. Ultimately, the variance derived from a convenience sample is susceptible to such idiosyncrasies and may therefore fail to accurately represent the true variability of the wider population (Mercer et al. 2017). This has direct implications for its use as a standardizer in effect size calculations.

## 2 Sensitivity Analysis

Sensitivity analysis assesses the robustness of meta‐analytic results by examining how variations, such as in model parameters, affect outcomes. We propose a sensitivity analysis to determine how within‐study standard deviations influence individual SMDs and overall meta‐analytic effect sizes. Grounded in data harmonization, our approach addresses potential SMD distortions from inconsistent standardizers. By harmonizing relative variability across studies, as quantified by the CV, we evaluate if idiosyncratic sample standard deviations disproportionately impact the standardized treatment effect.

### 2.1 Quantifying Variability

The CV, first introduced by Pearson (1896), is a standardized, scale‐independent measure of dispersion relative to the mean (Hendricks and Robey 1936; Vangel 1996). Its primary utility lies in allowing direct comparison of the level of dispersion between different sample outcomes by quantifying an outcome's relative variability. The population CV, denoted by κ, is defined as the ratio of the population standard deviation (σ) to the absolute value of the population mean (μ), assuming μ≠0, as follows

(8) $$\kappa =\frac{\sigma }{\left|\mu \right|}.$$

In practice, κ is an unknown parameter that must be estimated from sample data. For each study included in the meta‐analysis, the sample CV is calculated. For an outcome variable Y within a given study, the sample CV is the ratio of its sample standard deviation ($SD_{Y}$) to the absolute value of its sample mean (${\overset{®}{Y}}_{Y}$). This is expressed as

(9) $$\text{CV}=\frac{SD_{Y}}{\left|{\overset{®}{Y}}_{Y}\right|}.$$

This calculated CV serves as an estimate of the true underlying (κ) for the population from which that particular study's sample was drawn. A crucial precondition for the meaningful interpretation and application of the CV is that the outcome variable Y must be measured on a ratio scale. This implies that the variable has a true, non‐arbitrary zero point and typically takes on non‐negative values. Variables measured on interval scales, which lack a true zero and can have negative values, are generally not suitable for CV analysis.

The use of the absolute value of the mean, $\left|{\overset{®}{Y}}_{Y}\right|$, in the denominator of Equation (9) ensures that the CV is a dimensionless quantity. This allows it to be interpreted as a proportion or, when multiplied by 100, as a percentage, facilitating comparisons across diverse measures. A practical issue arises if a study's sample mean ${\overset{®}{Y}}_{Y}$ is zero, in which case its CV is mathematically undefined. Furthermore, if ${\overset{®}{Y}}_{Y}$ is very close to zero and the data for that sample includes both positive and negative values, the resulting CV can be highly unstable and may not accurately reflect the relative population variability. While such scenarios are less common with the types of ratio‐scale outcome data often used in relevant meta‐analyses, if encountered, a minimal non‐zero value might be substituted for the mean in practice, or more critically, the suitability of using the CV for that specific study's data should be carefully re‐evaluated.

Interpreting the CV, a higher value indicates greater relative variability observed within that study's sample (i.e., the data points are more dispersed relative to their sample mean), whereas a lower CV suggests that the data points are clustered more closely around their sample mean. For example, consider two studies: one representing students' heights in centimeters and another in inches. By calculating the CV for both, we can assess which study has greater relative variability relative to its respective mean. Thus, the sample CV provides a natural, convenient, and standardized metric for representing and comparing the relative variability of outcomes from different studies, which is essential for our proposed sensitivity analysis framework in a meta‐analytic context.

### 2.2 Data Harmonization

Data harmonization, in a general sense, refers to the process of standardizing and integrating data from disparate sources to ensure compatibility and comparability for combined analysis (Adhikari et al. 2021). A common aspect of this involves transforming data onto a common scale. Such practices are prevalent in meta‐analysis. For instance, individual participant data meta‐analyses often combine data sets where outcomes were measured on different scales, necessitating harmonization of these outcomes to a standard scale before pooling the data (Stewart and Tierney 2002). Similarly, in aggregate participant data (APD) meta‐analyses, coded study characteristics intended for use as effect modifiers in moderator analyses may require harmonization to ensure consistent interpretation. However, conventional APD meta‐analyses of SMDs (i.e., standardized treatment effects) typically do not involve harmonization of the variances (or standard deviations). Our sensitivity analysis harmonizes within‐study standardizers using the CV. By establishing benchmarks from the CV distribution (e.g., quartiles), we recalculate SMDs under harmonized variability assumptions. This explores how initial standardizer variability impacts SMDs and overall meta‐analytic results, assessing sensitivity rather than replacing original SMDs.

### 2.3 Quartiles

Quartiles are fundamental descriptive statistics that divide an ordered data set into four equal parts, offering key insights into a variable's distribution and spread. For any variable, three primary quartiles are identified: the first quartile ($Q_{1}$) or 25th percentile, the second quartile ($Q_{2}$) or 50th percentile (the median), and the third quartile ($Q_{3}$) or 75th percentile. These demarcate points below which 25%, 50%, and 75% of the data respectively lie (Dodd 1938). In our sensitivity analysis framework, we apply this concept to the empirical distribution of sample CVs calculated from the primary studies. Partitioning this CV distribution via its quartiles provides a structured understanding of the observed range and central tendency of relative variability across the included studies. These CV quartiles serve as data‐driven benchmarks representing low, median, and high levels of relative variability. By systematically applying these benchmark CV values back to each study's mean to derive alternative, harmonized standard deviations, we can then compute corresponding alternative SMDs. This process enables a robust evaluation of how different, consistently applied baseline levels of relative variability might influence individual study effect sizes and overall meta‐analytic conclusions, thereby making quartiles essential to our sensitivity analysis.

## 3 HSMDs

Our current algorithm implementation computes new standard deviations for $Q_{1}$, $Q_{2}$, and $Q_{3}$. Three new plausible SMDs exist, one for each quartile. This sensitivity analysis allows for comparing the effect of within‐study variability on individual studies and the meta‐analytical results. Below, we demonstrate the implementation of the HSMD using examples.

The HSMD is a sensitivity analysis approach to assess how within‐study variability (i.e., the sample standard deviations used to compute SMDs) affects individual study effect sizes and overall meta‐analytic results. Within‐study variability can alter the magnitude of SMDs and influence heterogeneity measures (e.g., between‐study variance ${\hat{\tau }}^{2}$ and $I^{2}$ statistic), which quantify how true effect sizes vary among studies. We propose an algorithm to compute the HSMD by harmonizing within‐study standard deviations using the CV, as follows.

1. Collect data: For all available primary studies, collect the best available representation of the mean treatment effect of each study i (e.g., ${\hat{\beta }}_{1}^{\left(i\right)}$ or mean difference $M_{T}^{\left(i\right)}-M_{C}^{\left(i\right)}$) and, where reported, the descriptive statistics for treatment (T) and control (C) groups, including means (${\overset{®}{Y}}_{T}^{\left(i\right)}$, ${\overset{®}{Y}}_{C}^{\left(i\right)}$) and standard deviations ($SD_{T}^{\left(i\right)}$, $SD_{C}^{\left(i\right)}$). Crucially, this HSMD approach can accommodate studies that do not report standard deviations, as harmonized standard deviations can be imputed for them in a later step.
2. Calculate CVs: For each study i that reports the necessary means and standard deviations for the treatment and/or control groups, calculate the sample CV for each group using Equation (9) (i.e., $CV_{T}^{\left(i\right)}=SD_{T}^{\left(i\right)}/|{\overset{®}{Y}}_{T}^{\left(i\right)}|$ and $CV_{C}^{\left(i\right)}=SD_{C}^{\left(i\right)}/|{\overset{®}{Y}}_{C}^{\left(i\right)}|$). If only one group's data are available (e.g., the control group's CV may serve as a proxy for population variability when the intervention does not substantially alter variance), use that CV. The CV assumes normally distributed outcomes, consistent with assumptions for SMD estimation, and a single population (for multiple populations, CV estimates may reflect heterogeneity).
3. Determine of CV distribution quartiles: From the collection of all calculated sample CVs (e.g., pooling CV 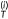 and CV 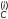 values, or using a prioritized set like control group CVs if theoretically justified), determine the empirical distribution of these CVs. Identify the key quartiles of this distribution: the 25th percentile ($Q_{1}($CV)), the 50th percentile or median ($Q_{2}($CV)), and the 75th percentile ($Q_{3}($CV)).
4. Estimate harmonized standard deviations: For each benchmark CV quartile ($Q_{k}($CV), where k∈{1,2,3}), estimate a harmonized standard deviation for each group (T and C) within each study i. This is achieved by rearranging the CV formula: $SD_{T,\text{harmonized},k}^{\left(i\right)}=|{\bar{Y}}_{T}^{\left(i\right)}|\times Q_{k}(\mathrm{CV})$ and $SD_{C,\text{harmonized},k}^{\left(i\right)}=|{\bar{Y}}_{C}^{\left(i\right)}|\times Q_{k}(\mathrm{CV})$. This step ensures that even studies initially lacking reported standard deviations will now have imputed standard deviations based on their reported means and the benchmark levels of relative variability from the overall set of studies.
5. Compute HSMDs: For each study i and for each CV quartile benchmark k, compute the HSMD. This involves calculating a new pooled standard deviation (${\hat{\sigma }}_{\text{pooled},\text{harmonized},k}^{\left(i\right)}$) using the harmonized group standard deviations ($SD_{T,\text{harmonized},k}^{\left(i\right)}$ and $SD_{C,\text{harmonized},k}^{\left(i\right)}$) and the original sample sizes. Then, the HSMD is calculated, for example, analogous to Cohen's d: HSMD
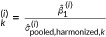
. Appropriate small‐sample corrections (e.g., for Hedges' g equivalent) should also be applied if used for the original SMDs.
6. Meta‐analyze HSMDs: For each set of HSMDs (i.e., one set for $Q_{1}($CV), one for $Q_{2}($CV), and one for $Q_{3}($CV)), fit a separate meta‐analytic model using the newly calculated HSMD values and their corresponding variances (calculated using standard formulas but with the harmonized standard deviations).

## 4 Illustrative HSMD Meta‐Analytic Applications

This section presents analyses of two example applications, Example I and Example II, which utilize sub‐datasets from data employed in prior meta‐analyses. The R functions and code (R Core Team 2021) used to generate these results and the corresponding plots are provided in the Supporting materials for reference.

### 4.1 Example I

The data for this first example originates from a systematic review and meta‐analysis on the effect of cognitive behavioral preventive school‐based interventions on depression in upper secondary education students (Kambara and Kira 2021). The original meta‐analysis included 18 studies and concluded that such interventions have a small‐to‐moderate effect in decreasing depression compared to control conditions. For this illustrative analysis, negative effect sizes indicate better performance of the treatment group relative to the control group (as lower scores on most depression measures signify lower anxiety). The data subset used here is available via the *Open Science Framework* (https://osf.io/cz9t6). As we are using a subset of the original data, the results presented are solely for illustrative purposes. Readers seeking to make substantive inferences about this intervention should consult the original article.

We begin by demonstrating the calculation of the CV and the subsequent derivation of HSMDs based on the distribution of CVs across all included studies. For instance, Kambara and Kira (2021) reported the following group means and standard deviations: treatment group (${\overset{®}{Y}}_{T}=12.46$, $s_{T}=10.38$) and control group (${\overset{®}{Y}}_{C}=21.90$, $s_{C}=10.71$). Using these values, the sample CVs for this study are

$$CV_{T}=\frac{10.38}{12.46}=0.8331\;\;\text{and}\;CV_{C}=\frac{10.71}{21.90}=0.4890.$$

After computing CVs for both treatment and control groups in all studies, we determine the quartiles of these CV distributions. For this data set, the quartiles for the treatment group CVs across all studies are $Q_{1_{T}}($CV)=0.2854, $Q_{2_{T}}($CV)=0.5067, and $Q_{3_{T}}($CV)=0.8360. For the control group CVs, they are $Q_{1_{C}}($CV)=0.2167, $Q_{2_{C}}($CV)=0.5258, and $Q_{3_{C}}($CV)=0.6481. These CV quartile values are then used to calculate harmonized standard deviations for each group within each study. Continuing with the Kambara and Kira (2021) study, the harmonized standard deviations for the treatment group, based on the treatment group CV quartiles, are

$$s_{Q1_{T}}=12.46\times 0.2854=3.5562,$$

$$s_{Q2_{T}}=12.46\times 0.5067=6.3139,$$

and

$$s_{Q3_{T}}=12.46\times 0.8360=10.4162.$$

For the control group, using the control group CV quartiles, the harmonized standard deviations are

$$s_{Q1_{C}}=21.90\times 0.2167=4.7454,$$

$$s_{Q2_{C}}=21.90\times 0.5258=11.5144,$$

and

$$s_{Q3_{C}}=21.90\times 0.6481=14.1935.$$

Comparing these harmonized standard deviations to the originally reported ones for the Kambara and Kira (2021) study offers insights. The reported $s_{T}$ (10.38) is very close to the harmonized standard deviation derived using the 75th percentile of treatment CVs ($s_{Q3_{T}}=10.4162$). This suggests that the observed sample variability in the treatment group of this particular study was relatively high compared to other treatment groups in the meta‐analysis. Conversely, the reported $s_{C}$ (10.71) is less than the harmonized standard deviation derived using the median of control CVs ($s_{Q2_{C}}=11.5144$), indicating that the control group exhibited somewhat lower relative sample variability than the typical control group.

Next, we compute the SMDs and HSMDs. For the Kambara and Kira (2021) study, using the originally reported means and standard deviations (and assuming $n_{T}=n_{C}=30$ for illustration, applying small‐sample bias correction for Hedges' g equivalent), the conventional SMD is calculated as (here, we used the SMD notation because the same steps can be taken using d or g).

$$\mathrm{SMD}=\frac{12.46-21.90}{\sqrt{\frac{(30-1)10.38^{2}+(30-1)10.71^{2}}{30+30-2}}}=\frac{-9.44}{10.5463}=-0.8951,$$

with var (SMD)=0.0714. The three HSMD values for this study, using the same mean difference but the corresponding harmonized pooled standard deviations are

$${\mathrm{HSMD}}_{25}=\frac{12.46-21.90}{\sqrt{\frac{(30-1)3.56^{2}+(30-1)4.75^{2}}{30+30-2}}}=\frac{-9.44}{4.1932}=-2.2513,$$

$${\mathrm{HSMD}}_{50}=\frac{12.46-21.90}{\sqrt{\frac{(30-1)6.31^{2}+(30-1)11.51^{2}}{30+30-2}}}=\frac{-9.44}{9.2857}=-1.0166,$$

and

$${\mathrm{HSMD}}_{75}=\frac{12.46-21.90}{\sqrt{\frac{(30-1)10.42^{2}+(30-1)14.19^{2}}{30+30-2}}}=\frac{-9.44}{12.4489}=-0.7583.$$

These calculations illustrate how altering the standardizer, based on different benchmarks of relative sample variability (CV quartiles), can substantially change the magnitude of an individual study's effect size. As expected, using a lower benchmark (from $Q_{1}($CV)) yields smaller harmonized standard deviations for the groups within a study; this leads to a smaller pooled standard deviation for that study and consequently amplifies its effect size. In contrast, a higher benchmark (from $Q_{3}($CV)) produces larger harmonized standard deviations, increasing the pooled standard deviation (the denominator of the effect size) and thus reducing the effect magnitude. This clear sensitivity of the calculated effect size to the underlying within‐study variance highlights the value of considering harmonization.

**Figure 1:**
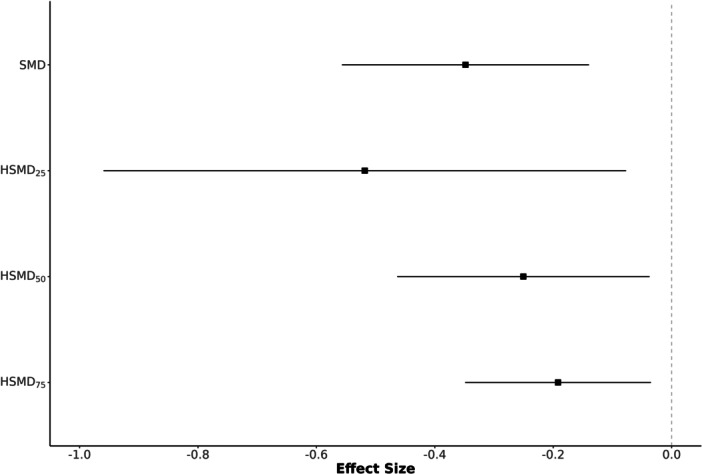
Estimated overall effect sizes between SMD and HSMD methods for Example 1.

**Figure 2:**
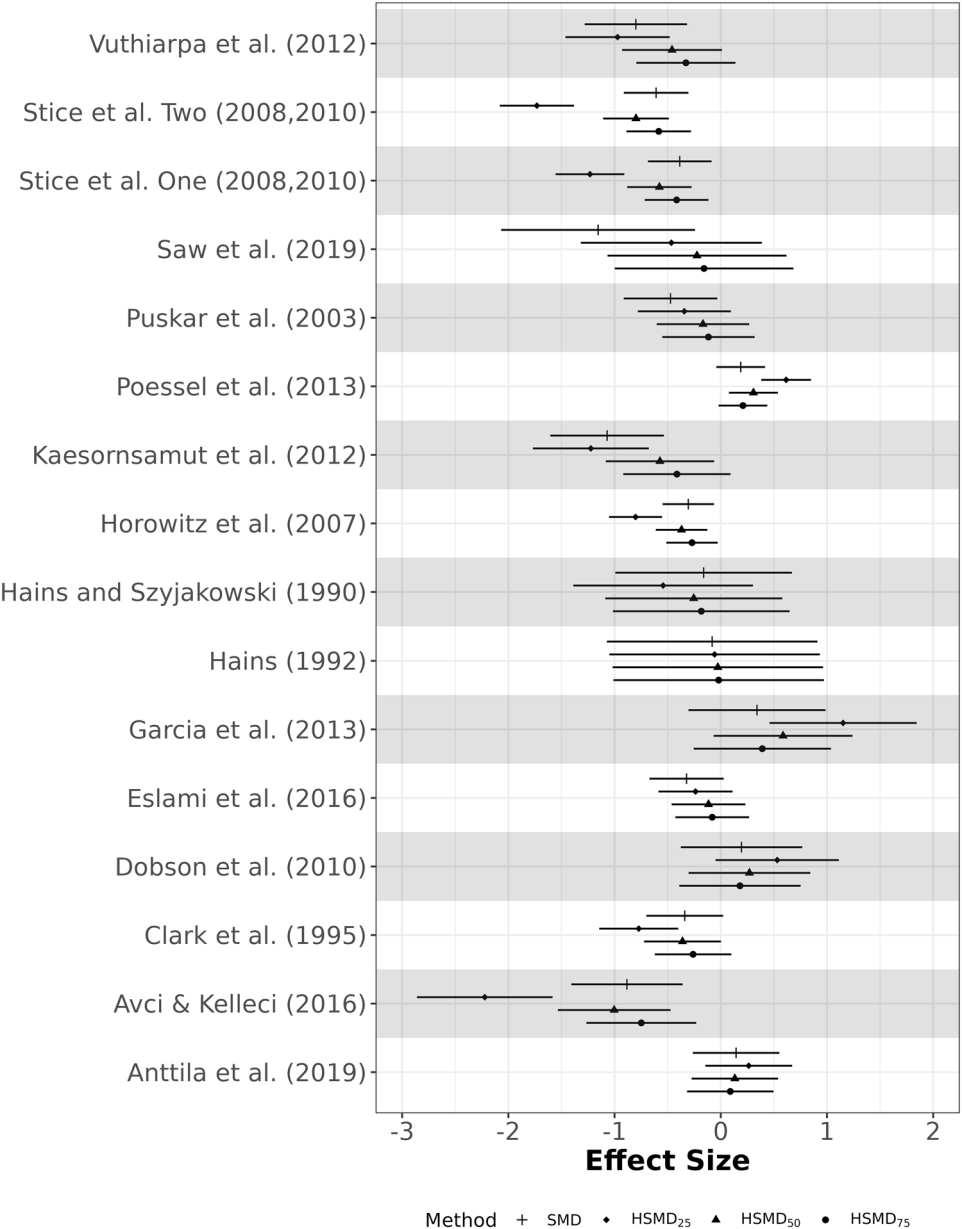
Estimated individual effect sizes between SMD and HSMD methods for Example 1.

We now turn to the overall meta‐analytic results. Table 1 presents overall meta‐analytic results under a random‐effects model, comparing the original SMD with HSMDs using quartile‐based standard deviations. Figure 1 illustrates differences in overall effect sizes and confidence intervals, while Figure 2 shows individual study effect sizes, emphasizing the impact of within‐study variance. Across studies, lower variance (e.g., HSMD_25_) amplifies effects but increases between‐study heterogeneity (${\hat{\tau }}^{2}$), while higher within‐study variance (e.g., HSMD_75_) reduces effect size magnitudes, shifting the overall effect toward zero.

**Table 1:** Overall meta‐analytical results comparing SMD and HSMD methods for Example 1.

Method	ES	SE	CI (lower)	CI (upper)	${\hat{\tau }}^{2}$	${\overset{®}{S}}_{\mathrm{within}}^{2}$	$I^{2}\;(\%)$
SMD	−0.3482	0.1064	−0.5568	−0.1397	0.1174	0.0419	73.70
${\mathrm{HSMD}}_{25}$	−0.5183	0.2251	−0.9595	−0.0770	0.7284	0.0455	94.12
${\mathrm{HSMD}}_{50}$	−0.2505	0.1087	−0.4635	−0.0374	0.1256	0.0419	75.00
${\mathrm{HSMD}}_{75}$	−0.1919	0.0800	−0.3487	−0.0351	0.0482	0.0413	53.84

Abbreviations: ${\hat{\tau }}^{2}$, estimated between‐studies variance (heterogeneity); CI (lower), lower bound of 95% confidence interval; CI (upper), upper bound of 95% confidence interval; ES, estimated overall effect size; HSMD, harmonized standardized mean difference; *I* ^2^, heterogeneity measure index; SE, standard error of estimate; SMD, standardized mean difference; ${\overset{®}{S}}_{\mathrm{within}}^{2}$, average within‐study sampling variance.

The variance of individual effect sizes (SMD or HSMD) is primarily driven by sample size, which remains constant across these analyses. Thus, as seen in Figure 2, the width of confidence intervals for individual study effects does not drastically change between SMD and HSMD variants. Consequently, the average within‐study sampling variance component (${\overset{®}{S}}_{\mathrm{within}}^{2}$ in Table 1) also remains relatively stable. Therefore, in this sensitivity analysis, changes in the estimated between‐studies variance (${\hat{\tau }}^{2}$, a measure of heterogeneity of true effects) and $I^{2}$ (percentage of total variation due to this heterogeneity) are primarily influenced by shifts in the point estimates of the effect sizes themselves.

Interpreting the results, HSMD_25_ yielded a pooled effect size estimate (ES = −0.5183) that is 48.85% larger in magnitude than the original pooled SMD (ES = −0.3482), due to lower within‐study variance. However, it also resulted in a 111.56% higher standard error (0.2251 vs. 0.1064). This increase in uncertainty led to a wider confidence interval [−0.9595, −0.0770], along with a markedly higher estimate of between‐study variance (${\hat{\tau }}^{2}=0.7284$ compared to 0.1174 in the original SMD analysis) and $I^{2}$ (94.12% vs. 73.70%). The substantial rise in ${\hat{\tau }}^{2}$ under this scenario suggests that if the true underlying relative variability in studies were consistently lower (i.e., if smaller standardizers were used), the dispersion of individual effect sizes would increase, leading to an inference of greater heterogeneity among true effects.

HSMD_50_ yielded a pooled effect size (ES = −0.2505), which is 28.06% smaller in magnitude than the original SMD. The standard error was only slightly higher (0.1087 vs. 0.1064, a 2.16% increase), and the confidence interval was slightly narrower [−0.4635, −0.0374], indicating comparable precision. The estimated between‐studies variance (${\hat{\tau }}^{2}=0.1256$) and $I^{2}$ (75%) were also slightly higher, suggesting that the median within‐study variance aligns well with the reported variances. If the median observed CV is considered a plausible representation of consistent relative sample variability, the HSMD_50_ results imply that the original SMD may have been influenced by studies with lower‐than‐median relative variability, potentially leading to a slight overestimation of the pooled effect size. Thus, HSMD_50_ may offer a more stable and bias‐adjusted estimate by accounting for standardizer choice.

HSMD_75_ produced the smallest pooled effect size magnitude (ES = −0.1919), a 44.89% reduction from the original SMD. This approach also yielded the narrowest confidence interval and substantially lower estimates of between‐studies variance (${\hat{\tau }}^{2}=0.0482$) and $I^{2}$ (53.84%). This demonstrates that systematically assuming higher relative sample variability across all studies (i.e., using larger standardizers) tends to attenuate individual effect sizes, leading to a smaller pooled effect and an inference of lower heterogeneity.

Considering HSMDs for this data set, the SMD pooled estimate (−0.3482) is situated between the HSMD_50_ (−0.2505) and HSMD_25_ (−0.5183) estimates. The substantial range observed in the pooled effect size estimates (from −0.1919 to −0.5183) and, most strikingly, in the estimated between‐studies variance (${\hat{\tau }}^{2}$ ranging from 0.0482 to 0.7284) across these HSMD analyses underscores a key conclusion: meta‐analytic inferences regarding both the overall effect magnitude and the degree of true effect heterogeneity are considerably sensitive to the variability of the standardizers reported in the primary studies. For this data set, it highlights that conclusions drawn from the SMD should be interpreted with caution, acknowledging the potential impact of idiosyncratic within‐study standard deviations. If a more stable estimate reflecting typical relative variability is desired, the HSMD_50_ results might offer a valuable point of comparison.

### 4.2 Example II

**Figure 3:**
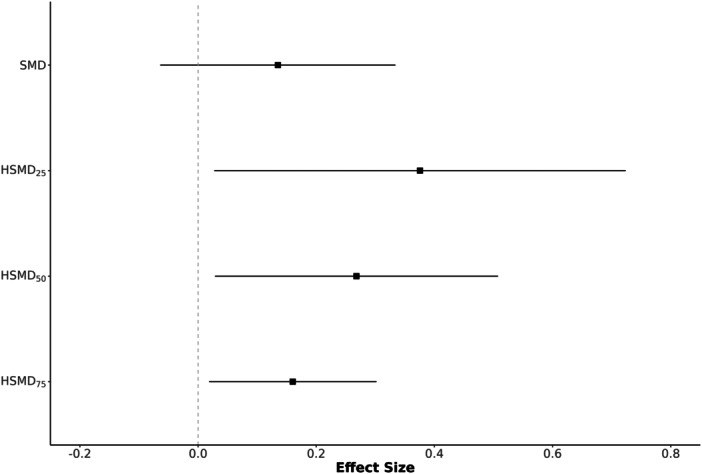
Estimated overall effect sizes between SMD and HSMD methods for Example 2.

**Figure 4:**
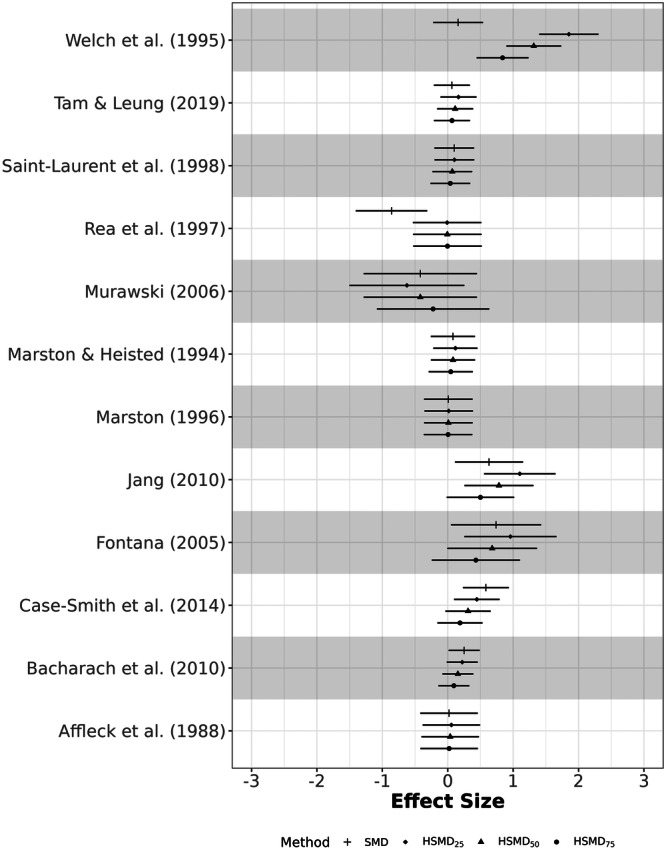
Estimated individual effect sizes between SMD and HSMD methods for Example 2.

The data for the second example are drawn from a systematic review and meta‐analysis investigating the impact of co‐teaching and collaborative instruction models on student achievement (Vembye et al. 2024). The original meta‐analysis encompassed 76 studies and 280 effect sizes. For this illustration, we utilized a subset of 12 studies. In this context, positive effect sizes indicate better performance by the treatment group. The data are available on the *Open Science Framework* (https://osf.io/fby7w/), and the results presented here are for illustrative purposes only, not for substantive inference. The summary of meta‐analytic results is presented in Table 2. The overall and individual effect sizes for each method are displayed in Figures 3 and 4, respectively. Figure 3 shows similar confidence interval widths for individual study effects across SMD and HSMD variants, with the average within‐study variance remaining stable as presented in Table 2.

**Table 2:** Overall meta‐analytical results comparing SMD and HSMD methods for Example 2.

Method	ES	SE	CI (lower)	CI (upper)	${\hat{\tau }}^{2}$	${\overset{®}{S}}_{\mathrm{within}}^{2}$	$I^{2}(\%)$
SMD	0.1351	0.1015	−0.0640	0.3341	0.0761	0.0371	67.19
${\mathrm{HSMD}}_{25}$	0.3756	0.1776	0.0275	0.7237	0.3211	0.0381	89.38
${\mathrm{HSMD}}_{50}$	0.2681	0.1222	0.0286	0.5077	0.1284	0.0375	77.42
${\mathrm{HSMD}}_{75}$	0.1603	0.0723	0.0187	0.3019	0.0215	0.0370	36.76

Abbreviations: ${\hat{\tau }}^{2}$, estimated between‐studies variance (heterogeneity); CI (lower), lower bound of 95% confidence interval; CI (upper), upper bound of 95% confidence interval; ES, estimated overall effect size; HSMD, harmonized standardized mean difference; *I* ^2^, heterogeneity measure index; SE, standard error of estimate; SMD, standardized mean difference; ${\overset{®}{S}}_{\mathrm{within}}^{2}$, average within‐study variability.

The SMD yielded an effect size of 0.1351 (SE = 0.1015, 95% CI [−0.0640, 0.3341]), with an estimated between‐studies variance (${\hat{\tau }}^{2}$) of 0.0761 and an $I^{2}$ of 67.19%. HSMD_25_ demonstrated the most substantial deviation, producing a 178% larger pooled effect size of 0.3756. This was accompanied by an increased standard error (0.1776), a wider confidence interval [0.0275, 0.7237], and notably higher heterogeneity (${\hat{\tau }}^{2}=0.3211$, $I^{2}=89.38\%$). HSMD_50_ resulted in an effect size of 0.2681, which is 98.4% larger than the SMD. The confidence interval [0.0286, 0.5077] was narrower than that of HSMD_25_ but wider than the SMDs. The standard error was 0.1222, and heterogeneity estimates (${\hat{\tau }}^{2}=0.1284$, $I^{2}=77.42\%$) were also higher than those from the SMD. Finally, HSMD_75_ produced an effect size of 0.1603, the closest to the SMD. This achieved a 28.8% decrease in standard error (0.0723) and the narrowest confidence interval [0.0187, 0.3019]. The estimated between‐studies variance was dramatically reduced to ${\hat{\tau }}^{2}=0.0215$, with $I^{2}$ decreasing to 36.76%.

Collectively, the HSMD sensitivity analysis reveals that the pooled effect size estimate is highly sensitive to the assumed level of relative within‐study variability, ranging from 0.1603 (HSMD_75_) to 0.3756 (HSMD_25_), compared to the SMD of 0.1351. The SMD aligns most closely with HSMD_75_, as shown in Figure 3, suggesting that high within‐study variance in the original data reduces heterogeneity but may underestimate the true effect. Notably, the estimated between‐study variance (${\hat{\tau }}^{2}$) varied substantially across scenarios (from 0.0215 to 0.3211), highlighting that, for this data set, conclusions about both the magnitude of the overall effect and the degree of heterogeneity are highly dependent on the standardizers used in the primary studies. Overall, the HSMD analyses suggest that the SMD may underestimate the true effect size in this context.

## 5 Alternative Approaches

While utilizing quartiles of the CV offers one plausible method to explore the impact of within‐study standardizer variability when synthesizing SMDs, it is not the sole option. Given that the fundamental role of the standard deviation in an SMD is to remove the scale of the original outcome measure, it is essential to acknowledge that alternative strategies for estimating or benchmarking population variability exist and warrant consideration. This section briefly discusses other approaches that could, in principle, be adapted or considered in the context of addressing variability in standardizers, though each comes with its own set of assumptions and practical challenges.

### 5.1 Established Norms

Normed measures provide standard deviations from large, representative samples, enabling comparison of individual scores to a reference group (Kolen 1996). In meta‐analyses, when studies use normed measures (e.g., standardized test scores), their standard deviations can standardize SMDs, potentially reducing within‐study variance variability. For example, if studies report math achievement using a normed test with a known standard deviation, this value can replace study‐specific standard deviations in the HSMD formula. However, this approach has limitations. If all studies report the same outcome, synthesis should use raw mean differences, as standardization is unnecessary. When outcomes differ, two challenges arise: (1) inconsistent use of normed measures across studies (e.g., different tests for achievement) and (2) variability in standard deviations among normed measures, which affects HSMD magnitudes and requires harmonization.

### 5.2 Robust Estimators of Variability

The minimum covariance determinant (MCD) is a robust multivariate method to estimate parameters of a normal distribution, less sensitive to outliers than traditional covariance estimators (Hubert et al. 2018; Rousseeuw and Driessen 1999). The MCD identifies a subset of data points that minimizes the determinant of the sample covariance matrix, providing reliable variance estimates. In meta‐analysis, where studies report different outcome measures, harmonizing scales using the CV enables MCD application. For instance, CV‐standardized outcomes across studies can be pooled, and the MCD can estimate a robust within‐study variance by focusing on a homogeneous subset of studies, mitigating the impact of outlying variances (e.g., from small or atypical studies). This approach enhances HSMD synthesis by providing stable standard deviations for the pooled variance term in the HSMD formula. However, MCD requires sufficient studies and assumes normality, limiting its use in sparse or heterogeneous data sets.

### 5.3 Post‐Stratification

Post‐stratification estimates population variance by stratifying studies into subgroups (e.g., by study design or population characteristics) and computing within‐stratum variances (Jagers 1986). These variances are combined to yield a precise overall estimate, reducing bias from subgroup variability. In meta‐analysis, post‐stratification can refine HSMD synthesis by accounting for within‐study variance differences across strata. For example, studies of co‐teaching (as in Example II) may be stratified by school level (elementary vs. secondary), with stratum‐specific standard deviations used in the HSMD formula. This approach improves accuracy over CV quartiles when subgroups have distinct variances. However, like other methods, scales must be harmonized using CVs before stratification, and sufficient studies per stratum are needed to avoid unstable estimates.

### 5.4 Bayesian Hierarchical Modeling

Bayesian hierarchical models can provide a framework for addressing standardizer variability by enabling principled pooling of information and regularization of variance estimates (Gelman et al. 2013; Spiegelhalter et al. 2004). In this approach, each study's true underlying standard deviation or CV can be modeled as originating from a common hyperdistribution, often using weakly informative priors. This structure facilitates partial pooling, shrinking noisy or extreme study‐specific variability estimates towards a more stable, overall mean. The resulting posterior mean estimates of these variability parameters then serve as regularized, data‐driven standardizers. Incorporating these into a full Bayesian meta‐analysis allows for comprehensive uncertainty propagation and yields (rich) posterior distributions for all parameters, including the pooled effect size. While conventional random‐effects models also address heterogeneity (Higgins et al. 2009), a fully Bayesian approach can handle multiple uncertainty sources, offering stabilized variance estimates and robust pooling. Though computationally intensive and requiring careful prior specification, Bayesian hierarchical modeling represents a powerful avenue for robustly harmonizing standardizers in SMD meta‐analyses.

## 6 Discussion and Conclusions

The foundational issue addressed by this study is the prevalent use of convenience samples in primary research. Non‐probabilistic sampling can lead to sample variances that unreliably estimate true population parameters, rendering the standard deviations used to compute SMDs potentially unrepresentative standardizers. This concern is magnified in meta‐analyses that synthesize findings from multiple such studies. Idiosyncratic within‐study sample variances, when used as standardizers in conventional SMD calculations, can unpredictably influence individual effect size estimates and, consequently, the overall meta‐analytic results. If such standardizer variability stems from factors such as sampling artifacts or study design peculiarities rather than true differences in population variability, the conclusions drawn from the meta‐analysis can be significantly affected. This underscores the critical need to systematically evaluate the impact of this standardizer variability.

The HSMD framework directly addresses this challenge by integrating data harmonization principles with sensitivity analysis to assess the robustness of meta‐analytic findings. The core of the HSMD approach is the harmonization of relative within‐study variability across studies, achieved by leveraging the CV. By establishing benchmarks for relative variability (e.g., using quartiles of the empirical CV distribution from the included studies) and then recalculating SMDs under these consistent variability assumptions, analysts can quantitatively gauge how much their original conclusions depend on the initial, study‐specific standardizers. For a methodologically sound comparison, it is imperative that HSMD analyses utilize the same meta‐analytic model structure (e.g., random‐effects model, adjustments for dependencies) as was employed for the original SMD synthesis.

A significant practical benefit of this harmonization‐based sensitivity analysis is HSMD's ability to incorporate studies that may have omitted reporting standard deviations. Once CV benchmarks are established, harmonized standard deviations can be imputed for these studies based on their reported means, thereby enabling their inclusion in the sensitivity assessment and potentially broadening the evidence base considered. When interpreting the results, if the original SMD pooled effect and associated heterogeneity estimates closely align with those from the HSMD_50_ analysis (which uses the median CV), it suggests that the overall conclusions may be relatively robust to standardizer variability. Conversely, substantial divergence between SMD results and the HSMD scenarios signals a critical sensitivity, indicating that the original findings were materially influenced by the specific standardizers used.

Given these considerations, we recommend that researchers conducting SMD meta‐analyses employ the HSMD framework as an integral sensitivity analysis. At a minimum, reporting the HSMD_50_ results alongside SMD findings, with a discussion of any notable discrepancies, would enhance the transparency and credibility of the meta‐analysis. Ideally, presenting the range of findings across different HSMD benchmarks (e.g., derived from $Q_{1}$, $Q_{2}$, and $Q_{3}$ of the CV distribution) as Supporting Information would offer a comprehensive illustration of the potential impact of standardizer variability.

While the HSMD offers a pragmatic approach for sensitivity analysis, it is important to acknowledge its limitations. The use of empirical CV quartiles, while providing practical harmonization benchmarks, is a heuristic approach; the robustness of these benchmarks can be influenced by the characteristics of the studies forming the CV distribution. Researchers should justify their decision on the source of CVs used to establish this distribution (e.g., control groups only, or both treatment and control groups), as this choice has implications for the resulting harmonized standard deviations. Furthermore, a core assumption in applying HSMD globally is that striving for consistent relative variability is a conceptually appropriate goal for the sensitivity analysis. In situations where a meta‐analysis combines studies from populations known to possess genuinely different underlying variabilities, applying a global harmonization might mask true heterogeneity, a critical consideration for interpretation.

In conclusion, the variability of standardizers is a critical, yet frequently unexamined, factor that can influence the outcomes of meta‐analyses using SMDs. The HSMD framework offers a transparent and systematic method for harmonizing this source of variability and thereby conducting a robust sensitivity analysis. By illuminating the extent to which meta‐analytic results depend on the idiosyncratic characteristics of individual study standardizers, this approach contributes to more cautious, nuanced, and ultimately more reliable interpretations of synthesized research evidence. Continued exploration and application of methods for critically evaluating standardizer variability, such as the HSMD framework, may prove beneficial for advancing meta‐analytic practice.

## Peer Review

The peer review history for this article is available at: https://www.webofscience.com/api/gateway/wos/peer-review/10.1002/cl2.70056.

## Supporting information

[Supporting] Standardized Mean Differences No So Standard After All.
