# Supplementary material for: Standardized Mean Differences: Not So Standard After All
Source: Campbell Syst Rev. 2025 Aug 17;21(3):e70056. doi: 10.1002/cl2.70056 (PMC12358740; doi:10.1002/cl2.70056)

Supplementary Material Standardized Mean Differences: Not So Standard After All

Juyoung Jung and Ariel M. Aloe

May 30, 2025

Table of Contents

[Illustrative HSMD Meta-Analytic Applications 1](#_Toc202502633)

[R Functions for Data Analysis 1](#_Toc202502634)

[Example 1 6](#_Toc202502635)

[Data Preparation for Example 1 6](#_Toc202502636)

[Analysis of $SMD$ and Harmonized Standardized Mean Differences (HSMD) 7](#_Toc202502637)

[(Table 1) Summary Statistics for $SMD$ and $HSMD$ 9](#_Toc202502638)

[(Figure 1) Plot of Overall $SMD$ and $HSMD$ 10](#_Toc202502639)

[(Figure 2) Plot of Individual $SMD$ and $HSMD$ by Study 11](#_Toc202502640)

[Example 2 13](#_Toc202502641)

[Data Preparation for Example 2 13](#_Toc202502642)

[Analysis of $SMD$ and Harmonized Standardized Mean Differences (HSMD) 14](#_Toc202502643)

[(Table 2) Summary Statistics for $SMD$ and $HSMD$ 17](#_Toc202502644)

[(Figure 3) Plot of Overall $SMD$ and $HSMD$ 17](#_Toc202502645)

[(Figure 4) Plot of Individual $SMD$ and $HSMD$ by Study 18](#_Toc202502646)

# Illustrative HSMD Meta-Analytic Applications

Below, we provide a series of functions written in the R language to reproduce the examples of the main manuscript.

## R Functions for Data Analysis

### 1. "SMD" function to calculate the Standardized Mean Difference.

## Input:
# mT, vT, nT: mean, variance, and sample size of the treatment group
# mC, vC, nC: mean, variance, and sample size of the control group

## Output:
# d: Cohen's d
# vd: variance of Cohen's d
# g: Hedges' g
# vg: variance of Hedges' g

SMD <- function(mT, vT, nT, mC, vC, nC) {
 # Calculates the standardized mean difference (Cohen's d)
 d <- (mT - mC)/(sqrt((vT*(nT -1) + vC*(nC-1)) / (nT + nC - 2)))
 # Estimates the variance of Cohen's d
 vd <- ((nT + nC) /(nT*nC)) + ((d^2) / (2*(nT + nC)))
 # Calculates a correction factor for small sample bias (Hedges' correction)
 J <- 1 - (3/ (4*(nT + nC - 2) -1))
 # Applies the correction factor to Cohen's d to get Hedges' g
 g <- J*d
 # Calculates the variance of Hedges' g
 vg <- J^2 * vd
 # Returns a matrix with four columns
 cbind(d, vd, g, vg)
}

### 2. "coefV" function to calculate the coefficient of variation.

## Input:
# mean: mean
# sd: standard deviation

## Output:
# If Mean is zero, it's approximately "sd/.001"
# If Mean is not zero, it's "sd/|mean|"

coefV <- function(mean, sd) {
 ifelse(mean == 0, sd/.001, sd/abs(mean))
}

### 3. "newSD" function to calculate a standard deviation based on a common coefficient of variation.

## Input:
# means: a vector of mean values
# cv_common: a common coefficient of variation

## Output:
# Multiplies the absolute value of each mean by the common CV

newSD <- function(means, cv_common) {
 abs(means)*cv_common
}

### 4. "aveWithin" function to calculate the average within-study variability.

## Input:
# s2: a vector of variances (squared standard deviations)

## Output:
# Compute the average within-study variability

aveWithin <- function(s2) {
 # Calculates weights (w) as the inverse of variances
 w <- 1/s2
 # Counts the number of variances (k).
 k <- length(s2)
 # Applies a formula to compute the average within-study variability
 (sum(w * (k-1))) / (sum(w)^2 - sum(w^2))
}

### 5. "HaSMD" function to perform a analysis involving Harmonized SMD (HSMD).

## Input:
# mT, sdT, nT: Mean, standard deviation, and sample size for the treatment group
# mC, sdC, nC: Mean, standard deviation, and sample size for the control group
# data: A data frame containing the above variables (mT, sdT, nT, mC, sdC, nC)

## Output:
# resAll (summary statistics for SMD and HSMD at each quartiles):
# est: estimated overall effect size
# se: standard error of estimate
# ci.lb: lower bound of 95% confidence interval
# ci.ub: upper bound of 95% confidence interval
# tau2: between-study variance
# avwW: average within-study variability
# I2: heterogeneity measure index

# A combined data frame of effect sizes and variances:
# ESdat: Traditional SMD results (d, vd, g, vg)
# ESdat25: HSMD results at 25th percentile (d25, vd25, g25, vg25)
# ESdat50: HSMD results at 50th percentile (d50, vd50, g50, vg50)
# ESdat75: HSMD results at 75th percentile (d75, vd75, g75, vg75)

# data (the original input data frame with additional columns):
# cvTp and cvCp: coefficients of variation for treatment and control groups
# SD1_25, SD1_50, SD1_75: standard deviations for the treatment group at each quartiles
# SD2_25, SD2_50, SD2_75: standard deviations for the control group at each quartiles

HaSMD <- function(mT, sdT, nT, mC, sdC, nC, data){

 # Converts input parameters to variable names
 mT <- deparse(substitute(mT))
 sdT <- deparse(substitute(sdT))
 nT <- deparse(substitute(nT))

 mC <- deparse(substitute(mC))
 sdC <- deparse(substitute(sdC))
 nC <- deparse(substitute(nC))

 # Extracts corresponding data
 mT <- data[[mT]]
 vT <- data[[sdT]]^2
 nT <- data[[nT]]

 mC <- data[[mC]]
 vC <- data[[sdC]]^2
 nC <- data[[nC]]

 ## Step1: Coefficient of Variation (CV) Calculation
 # Computes CV for both treatment and control groups using the "coefV" function
 data$cvTp <- coefV(mean = mT, sd = sqrt(vT))
 data$cvCp <- coefV(mean = mC, sd = sqrt(vC))

 ## Step2: Standard Deviation Calculation
 # Computes "SDs" based on 25th, 50th, and 75th percentiles of "CVs" for the treatment group
 data$SD1_25 <- newSD(mT, quantile(data$cvTp, na.rm = TRUE)[2])
 data$SD1_50 <- newSD(mT, quantile(data$cvTp, na.rm = TRUE)[3])
 data$SD1_75 <- newSD(mT, quantile(data$cvTp, na.rm = TRUE)[4])

 # Computes "SDs" based on 25th, 50th, and 75th percentiles of "CVs" for the control group
 data$SD2_25 <- newSD(mC, quantile(data$cvCp, na.rm = TRUE)[2])
 data$SD2_50 <- newSD(mC, quantile(data$cvCp, na.rm = TRUE)[3])
 data$SD2_75 <- newSD(mC, quantile(data$cvCp, na.rm = TRUE)[4])

 # Computes traditional SMD using the "SMD" function
 ESdat <- as.data.frame(SMD(mT = mT, vT = vT, nT = nT,
 mC = mC, vC = vC, nC = nC))
 names(ESdat) <- c('d', 'vd', 'g', 'vg')

 # Computes HSMD at 25th, 50th, and 75th percentiles using modified "SDs"
 ESdat25 <- as.data.frame(SMD(mT = mT, vT = data$SD1_25^2, nT = nT,
 mC = mC, vC = data$SD2_25^2, nC = nC))
 names(ESdat25) <- c('d25', 'vd25', 'g25', 'vg25')

 ESdat50 <- as.data.frame(SMD(mT = mT, vT = data$SD1_50^2, nT = nT,
 mC = mC, vC = data$SD2_50^2, nC = nC))
 names(ESdat50) <- c('d50', 'vd50', 'g50', 'vg50')

 ESdat75 <- as.data.frame(SMD(mT = mT, vT = data$SD1_75^2, nT = nT,
 mC = mC, vC = data$SD2_75^2, nC = nC))
 names(ESdat75) <- c('d75', 'vd75', 'g75', 'vg75')

 ## Performs meta-analysis using "metafor::rma.uni" for traditional "SMD" and "HSMDs"
 # For typical way
 res <- metafor::rma.uni(yi = ESdat[,1], vi = ESdat[,2])
 aveW <- aveWithin(ESdat[,2][complete.cases(ESdat[,2])])

 # For harmonized way
 res25 <- metafor::rma.uni(yi = ESdat25[, 1], vi = ESdat25[, 2])
 aveW25 <- aveWithin(ESdat25[, 2][complete.cases(ESdat25[, 2])])
 res50 <- metafor::rma.uni(yi = ESdat50[, 1], vi = ESdat50[, 2])
 aveW50 <- aveWithin(ESdat50[, 2][complete.cases(ESdat50[, 2])])
 res75 <- metafor::rma.uni(yi = ESdat75[, 1], vi = ESdat75[, 2])
 aveW75 <- aveWithin(ESdat75[, 2][complete.cases(ESdat75[, 2])])

 # Prepares summary statistics for traditional "SMD" and "HSMD" (25th, 50th, 75th percentiles)
 resT <- c('method' = 'SMD',
 'est' = round(res$b, 4),
 'se' = round(res$se, 4),
 "ci.lb" = round(res$ci.lb, 4),
 "ci.ub" = round(res$ci.ub, 4),
 'tau2' = round(res$tau2, 4),
 'avwW' = round(aveW, 4),
 'I2' = round(res$I2, 2))

 res25T <- c('method' = 'HSMD_25',
 'est' = round(res25$b, 4),
 'se' = round(res25$se, 4) ,
 "ci.lb" = round(res25$ci.lb, 4),
 "ci.ub" = round(res25$ci.ub, 4),
 'tau2' = round(res25$tau2, 4) ,
 'avwW' = round(aveW25, 4),
 'I2' = round(res25$I2, 2))

 res50T <- c('method' = 'HSMD_50',
 'est' = round(res50$b, 4),
 'se' = round(res50$se, 4) ,
 "ci.lb" = round(res50$ci.lb, 4),
 "ci.ub" = round(res50$ci.ub, 4),
 'tau2' = round(res50$tau2, 4) ,
 'avwW' = round(aveW50, 4),
 'I2' = round(res50$I2, 2))

 res75T <- c('method' = 'HSMD_75',
 'est' = round(res75$b, 4),
 'se' = round(res75$se, 4) ,
 "ci.lb" = round(res75$ci.lb, 4),
 "ci.ub" = round(res75$ci.ub, 4),
 'tau2' = round(res75$tau2, 4) ,
 'avwW' = round(aveW75, 4),
 'I2' = round(res75$I2, 2))

 # Returns a list containing
 resAll <- rbind(resT, res25T, res50T, res75T)
 return(list(resAll, cbind(ESdat, ESdat25, ESdat50, ESdat75), data))
}

## Example 1

### Data Preparation for Example 1

# Create data for example 1
data1 <- data.frame(
 Study = c("Saw et al. (2019)", "Vuthiarpa et al. (2012)", "Garcia et al. (2013)",
 "Avci & Kelleci (2016)", "Clark et al. (1995)", "Horowitz et al. (2007)",
 "Kaesornsamut et al. (2012)", "Dobson et al. (2010)", "Anttila et al. (2019)",
 "Eslami et al. (2016)", "Poessel et al. (2013)", "Stice et al. One (2008,2010)",
 "Stice et al. Two (2008,2010)", "Hains and Szyjakowski (1990)", "Hains (1992)",
 "Puskar et al. (2003)"),
 mT = c(68.90, 15.83, 12.96, 12.46, 17.88, 16.68, 16.33, 13.84, 7.50, 12.25, 14.55,
 10.71, 10.71, 6.67, 68.44, 63.85),
 sdT = c(10.29, 4.91, 11.30, 10.38, 9.30, 10.80, 5.71, 10.93, 3.70, 2.38, 12.29,
 9.07, 9.07, 3.16, 13.09, 13.48),
 nT = c(10, 35, 17, 30, 52, 108, 30, 25, 50, 63, 140, 88, 88, 9, 6, 42),
 mC = c(77.80, 20.20, 9.49, 21.90, 21.67, 20.34, 22.20, 12.00, 7.00, 13.01, 12.41,
 14.55, 16.48, 7.67, 69.50, 69.68),
 sdC = c(1.75, 5.86, 8.60, 10.71, 12.30, 12.60, 5.10, 6.75, 3.10, 2.31, 10.46,
 10.68, 9.80, 7.35, 11.50, 10.60),
 nC = c(10, 35, 19, 30, 68, 169, 30, 21, 42, 63, 152, 85, 84, 12, 8, 38)
)

# Check the data
print(data1)

## Study mT sdT nT mC sdC nC
## 1 Saw et al. (2019) 68.90 10.29 10 77.80 1.75 10
## 2 Vuthiarpa et al. (2012) 15.83 4.91 35 20.20 5.86 35
## 3 Garcia et al. (2013) 12.96 11.30 17 9.49 8.60 19
## 4 Avci & Kelleci (2016) 12.46 10.38 30 21.90 10.71 30
## 5 Clark et al. (1995) 17.88 9.30 52 21.67 12.30 68
## 6 Horowitz et al. (2007) 16.68 10.80 108 20.34 12.60 169
## 7 Kaesornsamut et al. (2012) 16.33 5.71 30 22.20 5.10 30
## 8 Dobson et al. (2010) 13.84 10.93 25 12.00 6.75 21
## 9 Anttila et al. (2019) 7.50 3.70 50 7.00 3.10 42
## 10 Eslami et al. (2016) 12.25 2.38 63 13.01 2.31 63
## 11 Poessel et al. (2013) 14.55 12.29 140 12.41 10.46 152
## 12 Stice et al. One (2008,2010) 10.71 9.07 88 14.55 10.68 85
## 13 Stice et al. Two (2008,2010) 10.71 9.07 88 16.48 9.80 84
## 14 Hains and Szyjakowski (1990) 6.67 3.16 9 7.67 7.35 12
## 15 Hains (1992) 68.44 13.09 6 69.50 11.50 8
## 16 Puskar et al. (2003) 63.85 13.48 42 69.68 10.60 38

# Study: the names of the studies
# mT: the mean value for the treatment group
# sdT: the standard deviation for the treatment group
# nT: the sample size in the treatment group
# mC: the mean value for the control group
# sdC: the standard deviation for the control group
# nC: the sample size in the control group

### Analysis of $\boldsymbol{SMD}$ and Harmonized Standardized Mean Differences (HSMD)

# Apply "HaSMD" function to calculate Harmonized Standardized Mean Differences (HSMD)
hsmd1 <- HaSMD(mT = mT, sdT = sdT, nT = nT, mC = mC, sdC = sdC, nC = nC, data = data1)

# Check the results
print(hsmd1)

## [[1]]
## method est se ci.lb ci.ub tau2 avwW
## resT "SMD" "-0.3482" "0.1064" "-0.5568" "-0.1397" "0.1174" "0.0419"
## res25T "HSMD_25" "-0.5183" "0.2251" "-0.9595" "-0.077" "0.7284" "0.0455"
## res50T "HSMD_50" "-0.2505" "0.1087" "-0.4635" "-0.0374" "0.1256" "0.0419"
## res75T "HSMD_75" "-0.1919" "0.08" "-0.3487" "-0.0351" "0.0482" "0.0413"
## I2
## resT "73.7"
## res25T "94.12"
## res50T "75"
## res75T "53.84"
##
## [[2]]
## d vd g vg d25 vd25
## 1 -1.20586354 0.23635267 -1.15491156 0.21680118 -0.48593445 0.20590331
## 2 -0.80837472 0.06181050 -0.79942592 0.06044957 -0.98244359 0.06403711
## 3 0.34831791 0.11314018 0.34057751 0.10816760 1.17798904 0.13072814
## 4 -0.89510143 0.07334339 -0.88347674 0.07145074 -2.25127453 0.10890197
## 5 -0.34134003 0.03442212 -0.33916589 0.03398502 -0.77729318 0.03645409
## 6 -0.30673940 0.01534625 -0.30590208 0.01526259 -0.80473374 0.01634537
## 7 -1.08430647 0.07646434 -1.07022457 0.07449115 -1.23939962 0.07946760
## 8 0.19855908 0.08804759 0.19515521 0.08505469 0.54061719 0.09079586
## 9 0.14536276 0.04392436 0.14414803 0.04319332 0.26564443 0.04419304
## 10 -0.32405772 0.03216275 -0.32209374 0.03177408 -0.23931225 0.03197330
## 11 0.18814942 0.01378242 0.18766240 0.01371116 0.61697401 0.01437361
## 12 -0.38812692 0.02356373 -0.38642211 0.02335718 -1.23700169 0.02755081
## 13 -0.61165180 0.02435595 -0.60894936 0.02414120 -1.73911617 0.03206062
## 14 -0.16788204 0.19511550 -0.16116676 0.17981845 -0.56568177 0.20206339
## 15 -0.08697268 0.29193682 -0.08142124 0.25585771 -0.06210885 0.29180443
## 16 -0.47791153 0.05155281 -0.47330146 0.05056302 -0.34674185 0.05087675
## g25 vg25 d50 vd50 g50 vg50
## 1 -0.46540201 0.18887064 -0.23404044 0.20136937 -0.22415140 0.18471176
## 2 -0.97156783 0.06262716 -0.46433901 0.05868293 -0.45919873 0.05739087
## 3 1.15181150 0.12498256 0.59973857 0.11645075 0.58641105 0.11133267
## 4 -2.22203720 0.10609172 -1.01662357 0.07527936 -1.00342067 0.07333675
## 5 -0.77234227 0.03599118 -0.36270684 0.03448480 -0.36039661 0.03404690
## 6 -0.80253702 0.01625625 -0.37035100 0.01542400 -0.36934003 0.01533991
## 7 -1.22330352 0.07741690 -0.58020346 0.06947197 -0.57266835 0.06767922
## 8 0.53134947 0.08770954 0.27452891 0.08843824 0.26982270 0.08543207
## 9 0.26342456 0.04345752 0.13346629 0.04390633 0.13235097 0.04317559
## 10 -0.23786187 0.03158691 -0.11635830 0.03179976 -0.11565310 0.03141548
## 11 0.61537701 0.01429930 0.30816706 0.01388442 0.30736939 0.01381263
## 12 -1.23156830 0.02730931 -0.58066772 0.02410284 -0.57811720 0.02389156
## 13 -1.73143230 0.03177794 -0.80225161 0.02513935 -0.79870706 0.02491770
## 14 -0.54305450 0.18622162 -0.26513847 0.19611822 -0.25453293 0.18074255
## 15 -0.05814446 0.25574169 -0.02962737 0.29169802 -0.02773626 0.25564842
## 16 -0.34339707 0.04989994 -0.16922126 0.05030429 -0.16758891 0.04933847
## d75 vd75 g75 vg75
## 1 -0.16442058 0.20067585 -0.15747323 0.18407561
## 2 -0.33199737 0.05793016 -0.32832212 0.05665467
## 3 0.39997731 0.11367708 0.39108893 0.10868090
## 4 -0.75829656 0.07145845 -0.74844856 0.06961444
## 5 -0.26243776 0.03422362 -0.26076619 0.03378904
## 6 -0.27143526 0.01530941 -0.27069430 0.01522594
## 7 -0.41855323 0.06812656 -0.41311747 0.06636853
## 8 0.18353413 0.08798519 0.18038783 0.08499441
## 9 0.09012547 0.04385367 0.08937233 0.04312380
## 10 -0.08102400 0.03177208 -0.08053294 0.03138813
## 11 0.20924582 0.01379678 0.20870420 0.01372545
## 12 -0.41782302 0.02363290 -0.41598778 0.02342574
## 13 -0.58669989 0.02426903 -0.58410770 0.02405505
## 14 -0.19105068 0.19531350 -0.18340865 0.18000092
## 15 -0.02100164 0.29168242 -0.01966111 0.25563475
## 16 -0.11742478 0.05021149 -0.11629206 0.04924745
##
## [[3]]
## Study mT sdT nT mC sdC nC cvTp
## 1 Saw et al. (2019) 68.90 10.29 10 77.80 1.75 10 0.1493469
## 2 Vuthiarpa et al. (2012) 15.83 4.91 35 20.20 5.86 35 0.3101706
## 3 Garcia et al. (2013) 12.96 11.30 17 9.49 8.60 19 0.8719136
## 4 Avci & Kelleci (2016) 12.46 10.38 30 21.90 10.71 30 0.8330658
## 5 Clark et al. (1995) 17.88 9.30 52 21.67 12.30 68 0.5201342
## 6 Horowitz et al. (2007) 16.68 10.80 108 20.34 12.60 169 0.6474820
## 7 Kaesornsamut et al. (2012) 16.33 5.71 30 22.20 5.10 30 0.3496632
## 8 Dobson et al. (2010) 13.84 10.93 25 12.00 6.75 21 0.7897399
## 9 Anttila et al. (2019) 7.50 3.70 50 7.00 3.10 42 0.4933333
## 10 Eslami et al. (2016) 12.25 2.38 63 13.01 2.31 63 0.1942857
## 11 Poessel et al. (2013) 14.55 12.29 140 12.41 10.46 152 0.8446735
## 12 Stice et al. One (2008,2010) 10.71 9.07 88 14.55 10.68 85 0.8468721
## 13 Stice et al. Two (2008,2010) 10.71 9.07 88 16.48 9.80 84 0.8468721
## 14 Hains and Szyjakowski (1990) 6.67 3.16 9 7.67 7.35 12 0.4737631
## 15 Hains (1992) 68.44 13.09 6 69.50 11.50 8 0.1912624
## 16 Puskar et al. (2003) 63.85 13.48 42 69.68 10.60 38 0.2111198
## cvCp SD1_25 SD1_50 SD1_75 SD2_25 SD2_50 SD2_75
## 1 0.02249357 19.664603 34.913957 57.598177 16.858189 40.904949 50.422719
## 2 0.29009901 4.518007 8.021596 13.233369 4.377062 10.620565 13.091760
## 3 0.90621707 3.698886 6.567270 10.834142 2.056352 4.989562 6.150535
## 4 0.48904110 3.556182 6.313903 10.416158 4.745428 11.514375 14.193542
## 5 0.56760498 5.103093 9.060400 14.947103 4.695591 11.393448 14.044477
## 6 0.61946903 4.760603 8.452319 13.943942 4.407398 10.694173 13.182495
## 7 0.22972973 4.660711 8.274963 13.651353 4.810434 11.672106 14.387974
## 8 0.56250000 3.950045 7.013196 11.569794 2.600235 6.309247 7.777283
## 9 0.44285714 2.140559 3.800503 6.269758 1.516804 3.680394 4.536748
## 10 0.17755573 3.496246 6.207489 10.240605 2.819088 6.840275 8.431871
## 11 0.84286865 4.152685 7.372977 12.163331 2.689076 6.524813 8.043007
## 12 0.73402062 3.056718 5.427119 8.953215 3.152785 7.649961 9.429956
## 13 0.59466019 3.056718 5.427119 8.953215 3.570989 8.664699 10.680802
## 14 0.95827901 1.903671 3.379914 5.575905 1.661983 4.032660 4.970980
## 15 0.16546763 19.533315 34.680860 57.213632 15.059693 36.541053 45.043431
## 16 0.15212400 18.223293 32.354952 53.376540 15.098696 36.635692 45.160091

### (Table 1) Summary Statistics for $\boldsymbol{SMD}$ and $\boldsymbol{HSMD}$

# Print the results
print(as.data.frame(hsmd1[[1]]))

## method est se ci.lb ci.ub tau2 avwW I2
## resT SMD -0.3482 0.1064 -0.5568 -0.1397 0.1174 0.0419 73.7
## res25T HSMD_25 -0.5183 0.2251 -0.9595 -0.077 0.7284 0.0455 94.12
## res50T HSMD_50 -0.2505 0.1087 -0.4635 -0.0374 0.1256 0.0419 75
## res75T HSMD_75 -0.1919 0.08 -0.3487 -0.0351 0.0482 0.0413 53.84

### (Figure 1) Plot of Overall $\boldsymbol{SMD}$ and $\boldsymbol{HSMD}$

# Prepare data for the overall "SMD" and "HSMD" plot
example1 <- as.data.frame(hsmd1[[1]])
example1$method <- factor(example1$method, levels = c("HSMD_75", "HSMD_50", "HSMD_25", "SMD"))
example1 <- cbind(example1[1], sapply(example1[-1], as.numeric))
print(example1)

# Create plot for overall "SMD" and "HSMD" plot
overallPLot1 <- ggplot(example1, aes(y = method)) +
 geom_point(aes(x = est), shape = 15, size = 3) +
 geom_linerange(aes(xmin = ci.lb, xmax = ci.ub)) +
 geom_vline(xintercept = 0, linetype = "dashed", color = "grey") +
 labs(x = "Effect Size", y = "", group = "Method") +
 theme_classic() +
 scale_x_continuous(limits = c(-1.00, 0.00), breaks = seq(-1.00, 0.00, by = 0.2)) +
 scale_y_discrete(labels = c("HSMD_75" = expression(HSMD[75]),
 "HSMD_50" = expression(HSMD[50]),
 "HSMD_25" = expression(HSMD[25]),
 "SMD" = "SMD")) +
 theme(axis.text = element_text(size = 11),
 axis.title.x = element_text(size = 11, face = "bold"),
 axis.title.y = element_text(size = 11))

# Display the plot
print(overallPLot1)


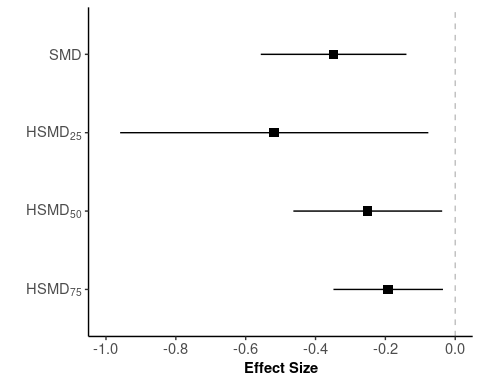


### (Figure 2) Plot of Individual $\boldsymbol{SMD}$ and $\boldsymbol{HSMD}$ by Study

# Extract columns with "g" in their names from "hsmd1[]" and remove rows with missing values.
hsmd1_g_df <- hsmd1[[2]][, grep("[g]", names(hsmd1[[2]]), value = TRUE) ]
hsmd1_g_df <- hsmd1_g_df[complete.cases(hsmd1_g_df), ]

# Create a list of data frames, each containing two columns ("g" and "vg")
hsmd1_g_list <- lapply(seq(1, ncol(hsmd1_g_df), by = 2), function(i)
 hsmd1_g_df[i: pmin((i+1), ncol(hsmd1_g_df))])

# Create an "ID" vector and combine it with the list of data frames
ID <- c("SMD","HSMD25", "HSMD50", "HSMD75")
hsmd1_g_list2 <- mapply(cbind, hsmd1_g_list, "Method" = ID, SIMPLIFY = F)

# Add the "Study" names to each data frame in the list
Study <- data1$Study
hsmd1_g_list2 <- lapply(hsmd1_g_list2, function(x) cbind(x, Study))

# Combine all data frames in the list into a single data frame
hsmd1_g_df2 <- data.table::rbindlist(hsmd1_g_list2, fill = FALSE)

# Calculate confidence intervals for each effect size
hsmd1_g_df2$conf.low <- hsmd1_g_df2$g - 1.96 * sqrt(hsmd1_g_df2$vg)
hsmd1_g_df2$conf.high <- hsmd1_g_df2$g + 1.96 * sqrt(hsmd1_g_df2$vg)

# Create a data frame for alternating shaded backgrounds in the plot
shades <- data.frame(ymin = seq(1.5, length(unique(hsmd1_g_df2$Study))-.5, 2),
 ymax = seq(2.5, length(unique(hsmd1_g_df2$Study))+.5, 2),
 xmin = -Inf, xmax = Inf)

# Reorder the data frame by Method
hsmd1_g_df2$Method <- factor(hsmd1_g_df2$Method, levels = c("HSMD75", "HSMD50", "HSMD25", "SMD"))

# Create plot for individual "SMD" and "HSMD" plot
indivPLot1 <- ggplot(hsmd1_g_df2, aes(x = g, y = Study, group = Method)) +
 geom_point(aes(shape = Method), position = position_dodge(width = 0.75)) +
 geom_errorbarh(aes(xmin = conf.low, xmax = conf.high),
 position = position_dodge(width = 0.75), height = 0) +
 labs(x = "Effect Size", y = "", shape = "Method") +
 theme_bw() +
 geom_rect(inherit.aes = F, data = shades,
 mapping = aes(xmin = xmin, xmax = xmax,
 ymin = ymin, ymax = ymax), alpha = 0.2) +
 theme(legend.position = "bottom") +
 scale_x_continuous(limits = c(-3, 2), breaks = seq(-3, 2, by = 1)) +
 scale_shape_manual(values = c(3, 18, 17, 16),
 labels = c(expression(SMD),
 expression(HSMD[25]),
 expression(HSMD[50]),
 expression(HSMD[75])),
 breaks = c("SMD", "HSMD25", "HSMD50", "HSMD75")) +
 theme(axis.text = element_text(size = 11),
 axis.title.x = element_text(size = 11, face = "bold"),
 axis.title.y = element_text(size = 11))

# Display the plot
print(indivPLot1)


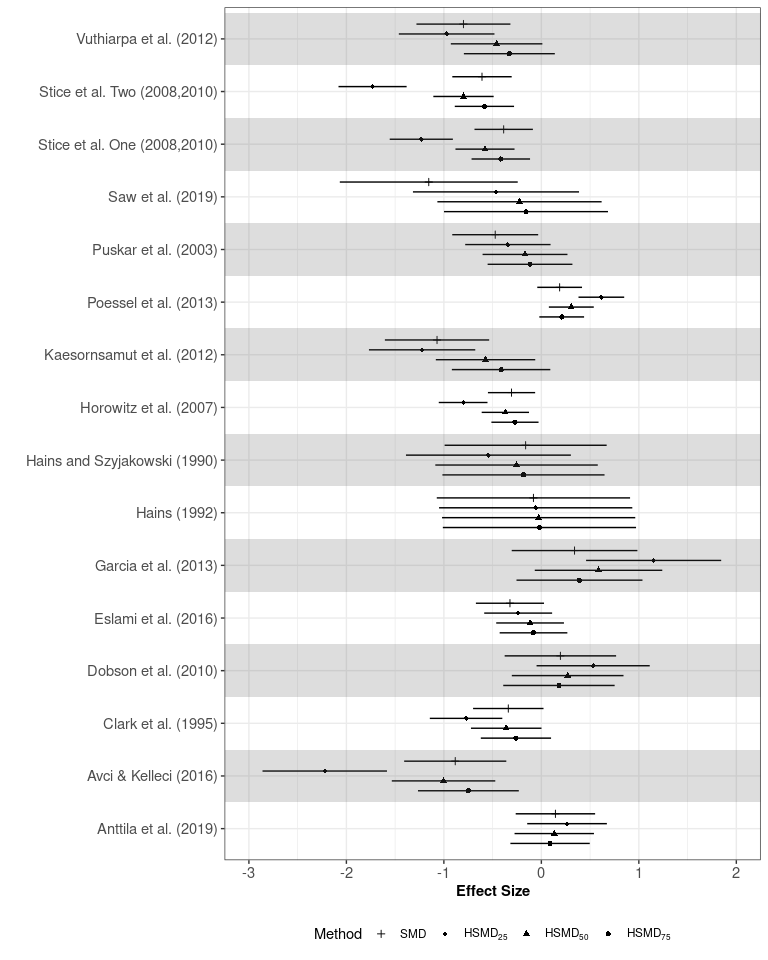


## Example 2

### Data Preparation for Example 2

# Create data for example 2
data2 <- data.frame(
 Study = c("Affleck et al. (1988)", "Bacharach et al. (2010)", "Case-Smith et al. (2014)",
 "Fontana (2005)", "Jang (2010)", "Marston & Heisted (1994)",
 "Marston (1996)", "Murawski (2006)", "Rea et al. (1997)",
 "Saint-Laurent et al. (1998)", "Tam & Leung (2019)", "Welch et al. (1995)"),
 mT = c(3.13, 17.20, 28.70, 11.20, 7.15, 9.60, 18.03, 2.08, -89.13, 16.50, 0.42, 2.33),
 sdT = c(7.770, 13.280, 17.150, 11.400, 8.300, 12.900, 33.210, 3.940, 0.969, 15.500, 0.800, 19.950),
 nT = c(39, 221, 77, 17, 28, 72, 33, 12, 36, 79, 110, 64),
 mC = c(2.970, 13.900, 18.500, 3.700, 1.950, 8.600, 17.770, 3.870, -88.322, 15.000, 0.360, -0.820),
 sdC = c(8.530, 12.760, 17.700, 8.000, 7.970, 12.100, 27.310, 4.330, 0.853, 14.500, 1.080, 19.330),
 nC = c(39, 99, 55, 16, 30, 62, 171, 8, 22, 86, 93, 44)
)

# Check the data
print(data2)

## Study mT sdT nT mC sdC nC
## 1 Affleck et al. (1988) 3.13 7.770 39 2.970 8.530 39
## 2 Bacharach et al. (2010) 17.20 13.280 221 13.900 12.760 99
## 3 Case-Smith et al. (2014) 28.70 17.150 77 18.500 17.700 55
## 4 Fontana (2005) 11.20 11.400 17 3.700 8.000 16
## 5 Jang (2010) 7.15 8.300 28 1.950 7.970 30
## 6 Marston & Heisted (1994) 9.60 12.900 72 8.600 12.100 62
## 7 Marston (1996) 18.03 33.210 33 17.770 27.310 171
## 8 Murawski (2006) 2.08 3.940 12 3.870 4.330 8
## 9 Rea et al. (1997) -89.13 0.969 36 -88.322 0.853 22
## 10 Saint-Laurent et al. (1998) 16.50 15.500 79 15.000 14.500 86
## 11 Tam & Leung (2019) 0.42 0.800 110 0.360 1.080 93
## 12 Welch et al. (1995) 2.33 19.950 64 -0.820 19.330 44

### Analysis of $\boldsymbol{SMD}$ and Harmonized Standardized Mean Differences (HSMD)

# Apply "HaSMD" function to calculate Harmonized Standardized Mean Differences
hsmd2 <- HaSMD(mT = mT, sdT = sdT, nT = nT, mC = mC, sdC = sdC, nC = nC, data = data2)

# Check the results
print(hsmd2)

## [[1]]
## method est se ci.lb ci.ub tau2 avwW I2
## resT "SMD" "0.1351" "0.1015" "-0.064" "0.3341" "0.0761" "0.0371" "67.19"
## res25T "HSMD_25" "0.3756" "0.1776" "0.0275" "0.7237" "0.3211" "0.0381" "89.38"
## res50T "HSMD_50" "0.2681" "0.1222" "0.0286" "0.5077" "0.1284" "0.0375" "77.42"
## res75T "HSMD_75" "0.1603" "0.0723" "0.0187" "0.3019" "0.0215" "0.037" "36.76"
##
## [[2]]
## d vd g vg d25 vd25
## 1 0.019610597 0.05128452 0.019416433 0.05027401 0.056404648 0.05130245
## 2 0.251487102 0.01472472 0.250893505 0.01465529 0.222363654 0.01470316
## 3 0.586862060 0.03247340 0.583469794 0.03209907 0.447252311 0.03192654
## 4 0.757444887 0.13001630 0.738970622 0.12375138 0.982115389 0.13593793
## 5 0.639545087 0.07257364 0.630941341 0.07063412 1.116587526 0.07979562
## 6 0.079766130 0.03004166 0.079312054 0.02970061 0.118089033 0.03006995
## 7 0.009178623 0.03615119 0.009144502 0.03588291 0.015306167 0.03615156
## 8 -0.437003028 0.21310762 -0.418538111 0.19547900 -0.651677215 0.21895041
## 9 -0.871438789 0.07977892 -0.859715397 0.07764684 -0.009856915 0.07323316
## 10 0.100087709 0.02431649 0.099626475 0.02409289 0.102529498 0.02431799
## 11 0.063926107 0.01985366 0.063687280 0.01970559 0.165011354 0.01991066
## 12 0.159891636 0.03847063 0.158757653 0.03792688 1.864907005 0.05445356
## g25 vg25 d50 vd50 g50 vg50
## 1 0.055846186 0.05029158 0.038537566 0.05129157 0.038156006 0.05028092
## 2 0.221838799 0.01463383 0.155568226 0.01466371 0.155201032 0.01459457
## 3 0.444667037 0.03155851 0.312821407 0.03153950 0.311013191 0.03117594
## 4 0.958161356 0.12938768 0.696704061 0.12867802 0.679711279 0.12247759
## 5 1.101566169 0.07766309 0.793723673 0.07447863 0.783045776 0.07248820
## 6 0.117416800 0.02972858 0.081167242 0.03004250 0.080705190 0.02970144
## 7 0.015249267 0.03588327 0.010147281 0.03615124 0.010109559 0.03588295
## 8 -0.624141558 0.20083847 -0.437170914 0.21311129 -0.418698904 0.19548237
## 9 -0.009724311 0.07127602 -0.006798745 0.07323272 -0.006707282 0.07127559
## 10 0.102057012 0.02409438 0.070051213 0.02430101 0.069728396 0.02407755
## 11 0.164394873 0.01976217 0.113687023 0.01987543 0.113262289 0.01972720
## 12 1.851680714 0.05368391 1.325090139 0.04648127 1.315692337 0.04582430
## d75 vd75 g75 vg75
## 1 0.021609711 0.05128504 0.021395753 0.05027453
## 2 0.093767829 0.01463964 0.093546504 0.01457061
## 3 0.188386410 0.03130326 0.187297471 0.03094242
## 4 0.441316332 0.12427444 0.430552519 0.11828620
## 5 0.506759666 0.07126146 0.499942272 0.06935701
## 6 0.046294152 0.03002592 0.046030618 0.02968504
## 7 0.005278190 0.03615105 0.005258569 0.03588277
## 8 -0.233767530 0.20969951 -0.223890028 0.19235282
## 9 -0.003917755 0.07323246 -0.003865050 0.07127533
## 10 0.039280156 0.02429081 0.039099141 0.02406745
## 11 0.065292277 0.01985410 0.065048345 0.01970603
## 12 0.844592117 0.04165475 0.838602102 0.04106600
##
## [[3]]
## Study mT sdT nT mC sdC nC cvTp
## 1 Affleck et al. (1988) 3.13 7.770 39 2.970 8.530 39 2.48242812
## 2 Bacharach et al. (2010) 17.20 13.280 221 13.900 12.760 99 0.77209302
## 3 Case-Smith et al. (2014) 28.70 17.150 77 18.500 17.700 55 0.59756098
## 4 Fontana (2005) 11.20 11.400 17 3.700 8.000 16 1.01785714
## 5 Jang (2010) 7.15 8.300 28 1.950 7.970 30 1.16083916
## 6 Marston & Heisted (1994) 9.60 12.900 72 8.600 12.100 62 1.34375000
## 7 Marston (1996) 18.03 33.210 33 17.770 27.310 171 1.84193012
## 8 Murawski (2006) 2.08 3.940 12 3.870 4.330 8 1.89423077
## 9 Rea et al. (1997) -89.13 0.969 36 -88.322 0.853 22 0.01087176
## 10 Saint-Laurent et al. (1998) 16.50 15.500 79 15.000 14.500 86 0.93939394
## 11 Tam & Leung (2019) 0.42 0.800 110 0.360 1.080 93 1.90476190
## 12 Welch et al. (1995) 2.33 19.950 64 -0.820 19.330 44 8.56223176
## cvCp SD1_25 SD1_50 SD1_75 SD2_25 SD2_50
## 1 2.872053872 2.8093901 3.9196820 5.9371829 2.8636419 4.3715974
## 2 0.917985612 15.4381818 21.5394668 32.6260531 13.4022297 20.4596645
## 3 0.956756757 25.7602220 35.9408545 54.4399840 17.8375000 27.2304887
## 4 2.162162162 10.0527696 14.0256993 21.2448718 3.5675000 5.4460977
## 5 4.087179487 6.4176163 8.9539063 13.5625744 1.8801689 2.8702407
## 6 1.406976744 8.6166596 12.0220280 18.2098901 8.2920270 12.6584975
## 7 1.536859876 16.1831638 22.5788713 34.2004499 17.1336419 26.1559884
## 8 1.118863049 1.8669429 2.6047727 3.9454762 3.7314122 5.6963239
## 9 0.009657843 80.0002992 111.6170160 169.0674485 85.1591176 130.0027690
## 10 0.966666667 14.8098837 20.6628606 31.2982486 14.4628378 22.0787747
## 11 3.000000000 0.3769789 0.5259637 0.7966827 0.3471081 0.5298906
## 12 23.573170732 2.0913351 2.9178464 4.4196921 0.7906351 1.2069730
## SD2_75
## 1 8.625000
## 2 40.366162
## 3 53.724747
## 4 10.744949
## 5 5.662879
## 6 24.974747
## 7 51.604798
## 8 11.238636
## 9 256.490657
## 10 43.560606
## 11 1.045455
## 12 2.381313

### (Table 2) Summary Statistics for $\boldsymbol{S}\boldsymbol{MD}$ and $\boldsymbol{HSMD}$

# Print the results
print(as.data.frame(hsmd2[[1]]))

## method est se ci.lb ci.ub tau2 avwW I2
## resT SMD 0.1351 0.1015 -0.064 0.3341 0.0761 0.0371 67.19
## res25T HSMD_25 0.3756 0.1776 0.0275 0.7237 0.3211 0.0381 89.38
## res50T HSMD_50 0.2681 0.1222 0.0286 0.5077 0.1284 0.0375 77.42
## res75T HSMD_75 0.1603 0.0723 0.0187 0.3019 0.0215 0.037 36.76

### (Figure 3) Plot of Overall $\boldsymbol{SMD}$ and $\boldsymbol{HSMD}$

# Prepare data for the overall "SMD" and "HSMD" plot
example2 <- as.data.frame(hsmd2[[1]])
example2$method <- factor(example2$method, levels = c("HSMD_75", "HSMD_50", "HSMD_25", "SMD"))
example2 <- cbind(example2[1], sapply(example2[-1], as.numeric))
print(example2)

# Create plot for overall "SMD" and "HSMD" plot
overallPLot2 <- ggplot(example2, aes(y = method)) +
 geom_point(aes(x = est), shape = 15, size = 3) +
 geom_linerange(aes(xmin = ci.lb, xmax = ci.ub)) +
 geom_vline(xintercept = 0, linetype = "dashed", color = "grey") +
 labs(x = "Effect Size", y = "", group = "Method") +
 theme_classic() +
 scale_x_continuous(limits = c(-0.2, 0.8), breaks = seq(-0.2, 0.8, by = 0.2)) +
 scale_y_discrete(labels = c("HSMD_75" = expression(HSMD[75]),
 "HSMD_50" = expression(HSMD[50]),
 "HSMD_25" = expression(HSMD[25]),
 "SMD" = "SMD")) +
 theme(axis.text = element_text(size = 11),
 axis.title.x = element_text(size = 11, face = "bold"),
 axis.title.y = element_text(size = 11))

# Display the plot
print(overallPLot2)


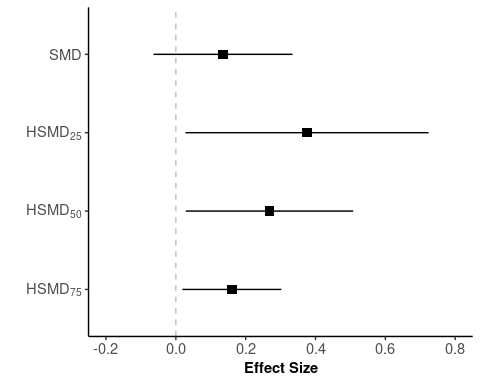


### (Figure 4) Plot of Individual $\boldsymbol{SMD}$ and $\boldsymbol{HSMD}$ by Study

# Extract columns with "g" in their names from "hsmd1[]" and remove rows with missing values.
hsmd2_g_df <- hsmd2[[2]][, grep("[g]", names(hsmd2[[2]]), value = TRUE) ]
hsmd2_g_df <- hsmd2_g_df[complete.cases(hsmd2_g_df), ]

# Create a list of data frames, each containing two columns ("g" and "vg")
hsmd2_g_list <- lapply(seq(1, ncol(hsmd2_g_df), by = 2), function(i)
 hsmd2_g_df[i: pmin((i+1), ncol(hsmd2_g_df))])

# Create an "ID" vector and combine it with the list of data frames
ID <- c("SMD","HSMD25", "HSMD50", "HSMD75")
hsmd2_g_list2 <- mapply(cbind, hsmd2_g_list, "Method" = ID, SIMPLIFY = F)

# Add the "Study" names to each data frame in the list
Study <- data2$Study
hsmd2_g_list2 <- lapply(hsmd2_g_list2, function(x) cbind(x, Study))

# Combine all data frames in the list into a single data frame
hsmd2_g_df2 <- data.table::rbindlist(hsmd2_g_list2, fill = FALSE)

# Calculate confidence intervals for each effect size
hsmd2_g_df2$conf.low <- hsmd2_g_df2$g - 1.96 * sqrt(hsmd2_g_df2$vg)
hsmd2_g_df2$conf.high <- hsmd2_g_df2$g + 1.96 * sqrt(hsmd2_g_df2$vg)

# Create a data frame for alternating shaded backgrounds in the plot
shades <- data.frame(ymin = seq(1.5, length(unique(hsmd2_g_df2$Study))-.5, 2),
 ymax = seq(2.5, length(unique(hsmd2_g_df2$Study))+.5, 2),
 xmin = -Inf, xmax = Inf)

# Reorder the data frame by Method
hsmd2_g_df2$Method <- factor(hsmd2_g_df2$Method, levels = c("HSMD75", "HSMD50", "HSMD25", "SMD"))

# Create plot for individual "SMD" and "HSMD" plot
indivPLot2 <- ggplot(hsmd2_g_df2, aes(x = g, y = Study, group = Method)) +
 geom_point(aes(shape = Method), position = position_dodge(width = 0.75)) +
 geom_errorbarh(aes(xmin = conf.low, xmax = conf.high),
 position = position_dodge(width = 0.75), height = 0) +
 labs(x = "Effect Size", y = "", shape = "Method") +
 theme_bw() +
 geom_rect(inherit.aes = F, data = shades,
 mapping = aes(xmin = xmin, xmax = xmax,
 ymin = ymin, ymax = ymax), alpha = 0.2) +
 theme(legend.position = "bottom") +
 scale_x_continuous(limits = c(-3, 3), breaks = seq(-3, 3, by = 1)) +
 scale_shape_manual(values = c(3, 18, 17, 16),
 labels = c(expression(SMD),
 expression(HSMD[25]),
 expression(HSMD[50]),
 expression(HSMD[75])),
 breaks = c("SMD", "HSMD25", "HSMD50", "HSMD75")) +
 theme(axis.text = element_text(size = 11),
 axis.title.x = element_text(size = 11, face = "bold"),
 axis.title.y = element_text(size = 11))

# Display the plot
print(indivPLot2)


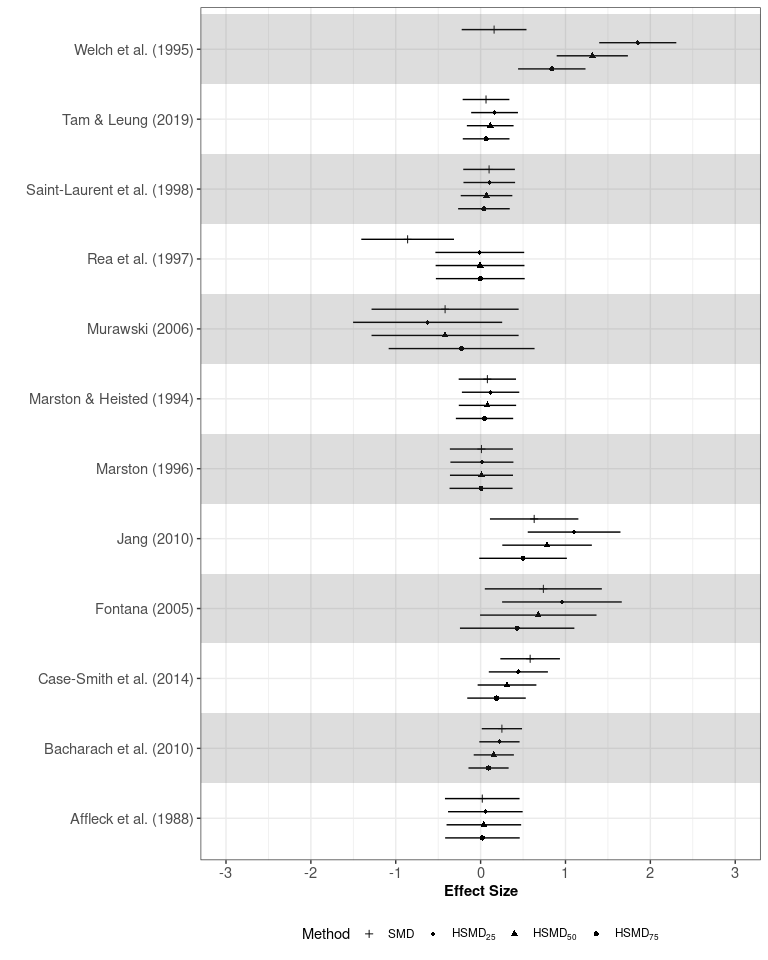

Supplement: Supplementary file 1 — [Supporting] Standardized Mean Differences No So Standard After All. [file CL2-21-e70056-s001.docx]
